# The effect of combining surgical or endoscopic bariatric interventions with anti-obesity medication or probiotics on weight loss: a narrative review

**DOI:** 10.3389/fendo.2026.1680182

**Published:** 2026-02-04

**Authors:** J. R. de Waal, M. Nieuwdorp, V. E. A. Gerdes

**Affiliations:** 1 Section of Vascular Medicine, Department of Internal Medicine, Amsterdam University Medical Centre, Amsterdam, Netherlands; 2 Department of Internal Medicine, Spaarne Gasthuis, Hoofddorp, Netherlands

**Keywords:** anti-obesity medication, bariatric surgery, endoscopic sleeve gastroplasty, gastric sleeve, insufficient weight loss, probiotics, Roux-en-Y gastric bypass, weight regain

## Abstract

Obesity is a multifactorial chronic disease associated with multiple health complications. While bariatric surgery and endoscopic sleeve gastroplasty (ESG) are effective treatments for severe obesity, patients may still experience challenges such as weight regain or inadequate weight loss. As a result, combining these interventions with adjunctive treatment such as anti-obesity medication (AOM) or probiotics has become an area of increasing clinical interest. This narrative review summarizes the current evidence on the effect of combining surgical or endoscopic bariatric interventions with AOM or probiotics. Available studies suggest that the combination of GLP-1 receptor agonists (GLP-1 RA) and ESG leads to more weight loss than ESG alone. In contrast, non-GLP-1 RA medication show less consistent benefits and generally lower weight loss. GLP-1 RAs and tirzepatide show clinically relevant short term weight loss in patients with weight regain or insufficient weight loss after bariatric surgery. Other AOM, such as topiramate, phentermine, orlistat and bupropion/naltrexone result in more modest and variable outcomes. Relatively small studies on probiotics and prebiotics in combination with bariatric surgery do not find a statistically significant difference in weight loss between probiotic and placebo groups. Although small benefits in waist circumference, liver enzymes and vitamin uptake are mentioned, the effects are modest and not consistent across studies. In conclusion, combining AOM, especially GLP-1 RAs, with surgical or endoscopic obesity treatments appears to enhance weight loss in patients with suboptimal outcomes. These findings support the use of AOM as an adjunct to bariatric procedures. In addition, additional adequately powered studies are needed to evaluate the role for probiotics in improving weight loss after bariatric surgery. Future research should focus on long-term outcomes, side effects, and identifying patient subgroups that may benefit most from combined therapy.

## Introduction

Obesity is a worldwide pandemic with an estimated one billion people affected (1). Obesity has a negative impact on multiple organ systems, leading to serious health complications. For example, obesity increases the risk of hypertension, dyslipidemia and type 2 diabetes, and as a result increased risk of cardiovascular events. Obesity can affect the respiratory system by reducing lung volumes, causing obstructive sleep apnea or have a negative impact on asthma. Obesity also affects the musculoskeletal system by increasing the risk of osteoarthritis. The gastrointestinal tract may be impacted in the case of metabolic dysfunction-associated steatotic liver disease and gastroesophageal reflux. Beyond physical health, obesity is also known to have a negative effect on the psychological well-being (2).

Bariatric surgery is the most effective therapy for severe obesity. It is associated with greater weight loss compared to combined life style interventions and pharmacologic treatment of obesity. Roux-en-Y Gastric Bypass (RYGB) and Sleeve Gastrectomy (SG) are the most performed interventions. Mean percentage weight loss long term after RYGB is 27.8 and 22.9 after SG (3). Other bariatric surgeries that are less frequently mentioned in this review are gastric banding, biliopancreatic diversion, gastric plication and Single Anastomosis Duodeno-Ileal bypass (SADI).

Another effective approach for weight loss is ESG, either as single intervention or in combination with anti-obesity medication (AOM). ESG is an emerging technique that reduces gastric volume by suturing the gastric body into a tubular configuration. By doing this, it mimics the restrictive effect of laparoscopic sleeve gastrectomy without anatomical resection. ESG as a single intervention reduces the total body weight by approximately 15.9% (4). Similar endoscopic procedures such as endoscopic gastric remodeling (EGR) lead to comparable results.

However, weight regain after initial normal weight loss, or insufficient weight loss after bariatric interventions, is a common problem. Most patients have some weight regain after the initial two years but still have sufficient long-term weight loss. A well-defined definition of abnormal weight regain after bariatric interventions is lacking. Nevertheless, a benchmark registry cohort of 18,403 patients shows that 12.2% achieve less than 20% weight loss compared to their starting weight. This was measured 5.2 years after surgery in context of insufficient weight loss or weight regain (5).

To address these issues, addition of pharmacologic treatment for obesity in those with insufficient weight loss after surgery or ESG is becoming more common. AOM mainly targets hormonal and neural pathways. These hormonal and neural pathways influence appetite, satiety, and glucose metabolism. GLP-1 receptor agonists (GLP-1RA), such as liraglutide and semaglutide, as well as the dual glucose-dependent insulinotropic polypeptide (GIP)/GLP-1 RA such as tirzepatide demonstrate a positive effect on bodyweight, glucose metabolism, and lipids. Other drugs such as topiramate alone or in combination with phentermine and bupropion/naltrexone show a clinically meaningful effect on weight regain. The combination of ESG in conjunction with AOM can increase weight loss to 25% (6, 7).

Obesity is associated with an altered gut microbiome composition. Therefore modifying the gut microbiome in patients with obesity by increasing the population of beneficial microorganisms has been identified as a potential strategy to increase weight loss and thereby lowering the chance of obesity related problems (8). Probiotics exhibit anti-obesity effects by regulating intestinal microflora and related metabolites, reducing insulin resistance, and enhancing satiety.

*Lactobacillus* and *Bifidobacterium* species have been used in animal models of obesity due to their low pathogenicity and strong resistance to antibiotics. Their application has resulted in varying degrees of weight and fat reduction. *Bifidobacterium* has been shown to reduce inflammation, insulin resistance, fat accumulation, and serum levels of cholesterol and triglycerides, primarily by decreasing intestinal permeability.

Furthermore, administering probiotics containing *Lactobacillus* strains to obese animals has effectively reduced body fat mass and improved both lipid distribution and blood glucose homeostasis, likely by stimulating fatty acid oxidation or inhibiting lipoprotein lipase activity (8).

It remains uncertain whether weight loss with pharmacological intervention and probiotics is the same after bariatric surgery and ESG compared to those without surgical intervention. It is important for patients and healthcare providers to understand how much weight loss they can expect from additional treatment after bariatric surgery or ESG. In this narrative review we discuss relevant articles investigating weight change in patients receiving a combination of AOM after or concomitant with bariatric interventions.

## Endoscopic sleeve gastroplasty and anti-obesity medication

We identified four studies that investigated the combined use of endoscopic bariatric procedures and AOM. In all studies, AOM was initiated directly or during the first year after the intervention. These studies included a total of 1,784 patients, of whom 381 received both endoscopic and pharmacological treatment, while the other 1,403 received endoscopic treatment alone. An overview of these articles can be found in **Table 1**.

**Table 1:** Primary endoscopic sleeve and anti-obesity medicine.

Study	Drug	Endoscopic intervention	Intervention	Patient profile	Time since surgery	Results
2025 Chung et al. Endoscopic sleeve gastroplasty combined with anti-obesity medication for better control of weight and diabetes	N=18 10 ESG monotherapy 8 ESG + semaglutide	18 ESG using the OverStitch Sx suturing system (Apollo Endosurgery Inc.)O	Retrospective study; ESG alone or in combination with semaglutide	Patients with obesity who underwent ESG	Semaglutide in the combination group started the second week after ESG	% TWL 6 months: ESG monotherapy: 15.32 ESG + semaglutide: 20.27 (p = 0.02)
2024 Jirapinyo et al. Efficacy of anti-obesity medication (AOM) and endoscopic gastric remodeling (EGR) Analysis of combination therapy with optimal timing and agents	N=208 65 EGR monotherapy 19 EGR + GLP-1RA 42 EGR + other AOM 21 EGR then AOM after six months 61 AOM then EGR after six months	208 EGR using the Overstitch endoscopic suturing device (Boston Scientific, Marlborough, Massachusetts, United States) or the Incisionless Operating Platform (USGI Medical, San Clemente, Calif, USA)	Retrospective review of prospectively collected data; EGR as monotherapy or in combination with AOM started within or after 6 months prior to or after EGR and concomitant moderate lifestyle modification after the procedure.	Patients with obesity, defined as BMI ≥30kg/m², or who are overweight, defined as BMI ≥ 27kg/m² with at least one obesity-related comorbidity such as hypertension, hyperlipidemia, diabetes/prediabetes and metabolic dysfunction-associated liver disease.	AOM within or after 6 months of EGR	% TWL 12 months EGR monotherapy: 17.3 ± 10.0 EGR + GLP1-RA: 23.7 ± 4.6 (p = 0.04) EGR + AOM: 18 ERG followed by AOM: 14.7 AOM followed by EGR: 12.0 ± 7.7 (p = 0.03)
2024 Gala et al. Outcomes of concomitant antiobesity medication use with endoscopic sleeve gastroplasty in clinical US settings	N=1.506 1.302 ESG monotherapy 58 GLP-1RA 146 Others (phentermine, phentermine-topiramate, bupropion-naltrexone, orlistat)	1.506 ESG using the Overstitch™ device (Apollo Endosurgery, Austin, Texas, USA)	Retrospective analysis; start anti-obesity medicine within 2 years after endoscopic sleeve gastroplasty	Patients who started anti- obesity medication after ESG. Reasons for starting are not mentioned.	<6 months after ESG 6–12 months after ESG >12 months after ESG	% TWL 24 months ESG monotherapy: 15.4 CI 14.2 – 16.6 ESG + GLP-1: 16.7 ± 11.9 ESG + other AOM: 14.2 ± 10.1 (p = not mentioned)
2021 Badurdeen et al. Endoscopic sleeve gastroplasty plus liraglutide versus endoscopic sleeve gastroplasty alone for weight loss	N=52 26 ESG monotherapy 26 ESG + liraglutide	56 ESG using the OverStitch; Apollo Endosurgery, Austin, Tex, USA)	Retrospective study; comparing the effect of additional liraglutide after ESG.	Patients aged >18 with a BMI >27kg/m² who were unable to achieve weight loss through intense diet and lifestyle modifications	In months: 5	% TWL 7 months ESG monotherapy: 20.51 ± 1.68 ESG + liraglutide: 24.72 ± 2.12 (p = <0.001)

The four studies had a similar design. All of the studies are retrospective cohort studies (9–12). The most common endoscopic procedure was ESG performed with the OverStitch device. Only Jirapinyo et al. used besides the OverStitch device also the Incisionless Operating Platform. The distribution between devices was not specified (11).

All studies compared the use of GLP-1 RAs, such as liraglutide and semaglutide in combination with ESG versus ESG monotherapy. Two studies also included non-GLP-1 RA medication like phentermine, topiramate, phentermine-topiramate, bupropion-naltrexone, bupropion and orlistat, used as monotherapy or combination therapy (11, 12). One study included patients who had initiated AOM prior to ESG (11). The other studies initiated AOM at varying intervals following ESG, ranging from two weeks (10) to more than 12 months after procedure (12). Study follow-up ranged from six months (10) to 24 months (12). Two studies investigated the start of semaglutide or liraglutide at a fixed time after intervention (9, 10). Two studies investigated the effectiveness of AOM at a non-fixed time after intervention (11, 12).

The overlapping outcome of these four studies (9–12) was the percentage of total body weight loss (%TWL), which varied across the different treatment strategies.

In the ESG monotherapy groups, %TWL ranged from 15.3% to 20.5%. In the ESG plus GLP-1 RA groups, higher weight loss outcomes were observed, with %TWL ranging from 16.7% to 24.7%. Chung et al. reported 20.27% TWL while this was 23.7% ± 4.6%, 16.7% ± 11.9%, and 24.72% ± 2.12% in the studies of Jirapinyo, Gala and Badurdeen respectively. One study also reported that addition of GLP-1 RA resulted in an additional reduction of HbA1C (1.2% ± 0.13% versus 1.4% ± 0.19% after six months in the ESG monotherapy and ESG combining AOM group respectively) (10).

For patients treated with ESG combined with non–GLP-1 anti-obesity medication, %TWL ranged from 14.2% to 18.0%. Jirapinyo et al. reported 18.0% but did not differentiate between different AOMs. Gala et al. reported 14.2% ± 10.1% and did not differentiate between types of non-GLP1 RAs either. These outcomes overlap with those observed in ESG monotherapy groups. Although the data suggest a modest benefit from the addition of non–GLP-1 AOM, the effect appears less consistent and less pronounced compared to addition of GLP-1 RAs.

The results suggest that combining ESG with GLP-1 RA therapy may result in superior weight loss compared to ESG alone or ESG combined with non–GLP-1 medications. These findings support the potential additive effect of GLP-1 RAs when initiated concurrently with endoscopic intervention.

A strength of the reviewed studies is that all four were conducted in clinical practice settings, increasing the external validity of the findings. However, several limitations must be considered. All included studies were retrospective cohort studies, introducing a risk of selection bias and limiting the ability to infer causality (9–12). In addition, none of the studies used randomization or blinding, and the timing of AOM initiation varied significantly, from two weeks to over 12 months post-ESG (8, 11). This heterogeneity may have influenced treatment effects and complicates direct comparison between studies. Furthermore, none of the studies included a standardized protocol for AOM type or dosage, and only limited information was available on patient adherence. Whether combination of ESG and AOM leads to additional and specific side effects is not reported. The limited follow-up duration in these studies (as short as seven months) also restricts conclusions on long-term efficacy. Future prospective studies on ESG combined with AOM with standardized initiation of AOM, defined medication protocols, side effects and longer follow-up are warranted to confirm these findings.

## Bariatric surgery and AOM

A total of 25 articles encompassing a total of 3,588 patients were identified. Characteristics and main results of these articles can be found in **Table 2**.

**Table 2:** Bariatric surgery and anti-obesity medication.

Study	Drug	Pre-intervention operation	Intervention	Patient profile	Time since surgery	Follow-up duration	Results
2025 Medhati et al. GLP-1RA in the Real World: 1-year Compliance and Outcomes of Semaglutide use in Patients With or Without Previous History of Bariatric Surgery For this review, only patients who had undergone bariatric surgery are included	271 Semaglutide	N=271 178 SG 80 RYGB 13 AGB	Retrospective series; semaglutide 0.25 to 2.4mg per week pending tolerance of dose escalation	Patients prescribed semaglutide for weight loss in adults with obesity.	Not mentioned	1 year	% TWL 1 year: RYGB: 13.6 ± 10.3 (p = 0.22) SG 11.4 ± 9.8 (p = 0.09) AGB not mentioned
2025 Lahooti et al A Randomized, Double-Blind, Two-Way Cross-over Study to Evaluate the Efficacy of Liraglutide Treatment in Patients Undergoing Transoral Outlet Reduction Endoscopy for Weight Regain Post Roux-en-Y Gastric Bypass	N=109 51 TORe + Liraglutide for 12 months followed by 12 months saline (LP) 58 TORe + saline for 12 months followed by 12 months liraglutide (PL)	109 RYGB	Randomized, double blind, two-way cross over study; TORe followed by saline or liraglutide. Crossover between the two after 12 months.	Patients with undefined weight regain after RYGB	Immediately after TORe or 12 months after TORe	24 months	% TWL 12 months: LP: 21.69 ± 1.64 PL: 14.37 ± 2.58 (p = <0.01) % TWL after 24 months LP: 15.74 ± 5.84 PL: 11.04 ± 4.36 (p = <0.01)
2025 Lofton et al. A randomized, double-blind, placebo-controlled trial of weight loss using liraglutide 3.0 mg for weight recurrence after Roux-en-Y gastric bypass	N=132 89 Liraglutide 43 Placebo	132 RYGB	Double-blind, placebo-controlled study; liraglutide 3.0mg/d or placebo with lifestyle counselling regularly for 56 weeks	Patients who achieved ≥25% TWL status-post-RYGB and regained ≥10% TWL after reaching nadir weight.	In months: 18–120 post RYGB	12 months	% TWL 12 months: Liraglutide: 8.8% Placebo: 1.1% Estimated treatment difference, liraglutide versus placebo (95% CI): -9.9 (6.3, 13.6)
2025 Bahdi et al. Revisional endoscopic sleeve gastroplasty versus semaglutide and tirzepatide for weight recidivism after sleeve gastrectomy	N=177 68 Prior SG + GLP1/GIP RA 22 Prior SG + R-ESG 87 Intact stomach + GLP1/GIP RA	N=177 90 SG 87 no pre intervention surgery	Retrospective study; semaglutide, tirzepatide, combination or revision endoscopic sleeve gastroplasy	Adult patients (≥18 years) with prior SG who were treated with weekly subcutaneous injections of semaglutide or tirzepatide or R-ESG for weight recidivism. Matched group based by age, gender and obesity class of patients with obesity and no prior gastric or bariatric surgery	In months: 52.4 ± 33.8 Prior SG + GLP1/GIP RA 74 ± 38.1 Prior SG + R-ESG	12 months	% TWL 12 months: Prior SG + R-ESG 13.4 ± 3.7 Prior SG + GLP1/GIP RA 9.2 ± 7 (p = 0.07) Intact stomach + GLP1/GIP RA 12.7 ± 6.8 (p = 0.03)
2025 Stoll et al. Tackling suboptimal clinical response after metabolic bariatric surgery: Impact of tirzepatide on weight loss and body composition	21 Tirzepatide	N=21 11 SG 10 RYGB	Retrospective cohort study; tirzepatide 2.5 mg/week first 4 weeks and increased to a maximum dose of 12.5mg/week after 6 months according to individual tolerability	Adult patients (≥18 years) with weight regain defined as continuous weight increase after an initial successful weight loss (defined as EWL]> 50 % 18-months post-MBS) and IWL defined as achieving a nadir weight with EWL < 50 % 18 months	In years: 7.2 ± 4.3	6 months	% TWL 6 months: 12.0 ± 3.4 (p = <0.01)
2024 Boger et al. Treatment with Antiobesity Drugs in Weight Regain After Bariatric Surgery: a Retrospective Cohort Study	N=80 5 Orlistat 5 Orlistat combination 18 Sibutramine/topiramate 15 Sibutramine 37 Topiramate	N=80 41 gastric bypass 29 ringed or banded gastric bypass 7 SG 2 biliopancreatic diversion 1 VBG	Retrospective cohort study; AOM for at least 2 years.	Patients >18 years with a history of bariatric surgery with weight regain of at least 5% after nadir despite dietary counselling and lifestyle changes.	Not mentioned	24 months	% TWL 24 months: Topiramate: 3.3 (p = 0.04) Topiramate/sibutramine: 5.9 (p = <0.01) Orlistat (in combination): 3.9 (p = 0.032)
2024 Jamal et al. Semaglutide and Tirzepatide for the Management of Weight Recurrence After Sleeve Gastrectomy: A Retrospective Cohort Study	N=115 70 Semaglutide 45 Tirzepatide	115 SG	Retrospective cohort study; Semaglutide or tirzepatide in increasing dose regimen starting at 0.25mg and 2.5mg respectively	Adult patients (≥18 years) with prior SG with a BMI greater than 30kg/m² or 27kg/m² with at least one obesity-related complication	In months: 71.8 ± 51.1	6 months	% weight change 6 months: Semaglutide: -10.3 ± 5.9 (p = <0.05) Tirzepatide: -15.5 ± 6.3 (p = <0.05)
2023 Hany et al. Boosting weight loss after conversional Roux-en-Y Gastric Bypass with liraglutide and placebo use. A double-blind-randomized controlled trial	N=64 35 Liraglutide 29 Placebo	64 cRYGB	Randomized double-blind placebo-controlled trial; daily injections of liraglutide or placebo for 6 months. After stopping liraglutide the patients were followed up for 6 more months	Patients who underwent conversional RYGB after SG with IWL defined as <50% of EWL, or weight regain defined as weight increase after achieving greater than 50% of EWL	In weeks: 6	12 months	% TWL 12 months: Liraglutide: 24.15 ± 2.35 Placebo: 22.70 ± 2.13 (p = <0.01)
2023 Mok et al. Safety and Efficacy of Liraglutide, 3.0 mg, Once Daily vs Placebo in Patients With Poor Weight Loss Following Metabolic Surgery: The BARI-OPTIMISE Randomized Clinical Trial	N=70 35 Liraglutide 35 Placebo	N=70 65 SG 5 RYGB	Randomized, placebo-controlled trial; liraglutide 3.0mg daily or placebo as adjunct to lifestyle intervention with a 500 kcal daily energy deficit	Patients with poor weight loss response defined as 20% or less TWL from the day of surgery following RYGB or SG	In months: 52.1 ± 33.4	6 months	KG weight loss 6 months: liraglutide: 9.49 ± 5.07 Placebo: 0.39 ± 3.88
2023 Murvelashivili et al. Effectiveness of semaglutide versus liraglutide for treating post-metabolic and bariatric surgery weight recurrence	N=207 92 Liraglutide 115 Semaglutide	N=207 104 VSG 60 RYGB 43 AGB	Retrospective analysis of electronic health records; treated with semaglutide 1.0mg or liraglutide 3.0mg alone or in combination with other anti-obesity medication	patients who sought help for post metabolic and bariatric surgery (MBS) weight recurrence	In years: 8.0 ± 6.44	12 months	% TWL 12 months: Semaglutide Overall 12.92 ± 1.17 (p = <0.01) AGB 8.64 ± 2.53 VSG 12.36 ± 1.86 RYGB 15.83 ± 2.48 Liraglutide Overall 8.77 ± 1.23 (p = <0.01) AGB 7.80 ± 2.80 VSG 8.28 ± 1.95 RYGB 8.26 ± 2.39
2023 Jirapinyo et al. Combining transoral outlet reduction with pharmacotherapy yields similar 1-year efficacy with improved safety compared with surgical revision for weight regain after Roux-en-Y gastric bypass (with videos)	N=796 145 combination 42% Topiramate 23% Phentermine/topiramate 21% phentermine 15% liraglutide 3% Dulaglutide 3% bupropion/naltrexone 3% bupropion 2% metformin 1% naltrexone 1% lorcaserin and TORe (CT). Pharmacotherapy started 4 ± 2 months before or after TORe. 211 pharmacotherapy (Ph) Exact numbers for medication are not given 363 TORe 77 surgical revision (SR)	796 RYGB	Retrospective comparative study of combination therapy with pharmoacotherapy and TORe, pharmacotherapy alone, TORe alone, surgical revision alone.	Patients with weight regain, defined as gaining at least 15% of the maximal weight initially lost with a body mass index of at least 30kg/m² or 27kg/m² with at least 1 obesity-related comorbidity.	In years: CT 12 ± 6 Ph 8 ± 5 TORe 11 ± 5 SR 7 ± 4	12 months	% TWL 12 months CT 15.2 ± 7.4 Ph 6.8 ± 8.2 (p = <0.01) TORe 8.7 ± 8.3 (p = <0.01) SR 16.4 ± 13.1 (p = 0.34)
2022 Dharmaratnam et al. Revisional Surgery or Pharmacotherapy for Insufficient Weight Loss and Weight Regain After Primary Bariatric Procedure: a Descriptive Study For this review, only the pharmacological treatment is included	N=150 11 Orlistat 112 Phentermine 31 Liraglutide 28 Topiramate 32 People had combination therapy but what combination is not mentioned	SG or RYGB, specific numbers are not mentioned	Retrospective cohort study; AOM after bariatric surgery	Patients with IWL defined as <20% TWL 18 months after bariatric surgery or WR <10% or >10% after NADIR	In months: IWL: 41.1 ± 26.2 WR <10%: 48.7 ± 24.9 WR ≥ 10%: 54.8 ± 28.7	Median follow up of 72 months with an interquartile range of 48 months	% total weight loss: IWL: 14.9 ± 3.7 WR <10%: 25.6 ± 5.0 WR ≥ 10%: 29.8 ± 5.7 (p = <0.01)
2022 Muratori et al. Efficacy of liraglutide 3.0 mg treatment on weight loss in patients with weight regain after bariatric surgery	62 Liraglutide	62 total 23 SG 17 RYGB 22 LGB	Retrospective observational study; liraglutide after weight regain post- bariatric surgery	Patients aged >18 whom started liraglutide when there was a 10-15% weight regain over the nadir obtained after any bariatric surgery	In months: 70.7 ± 43.7	variable	BMI drop during use: RYGB: 5.0 ± 6.4 LGB: 7.6 ± 5.8 SG 5.6 ± 6.9 (p = not mentioned)
2022 Elhag et al. Effectiveness and Safety of Liraglutide in Managing Inadequate Weight Loss and Weight Regain after Primary and Revisional Bariatric Surgery: Anthropometric and Cardiometabolic Outcomes	145 Liraglutide	145 total 110 primary SG 6 primary RYGB 3 primary AGB 14 revisional SG 11 revisional RYGB 1 revisional Single Anastomosis Duodeno-ileal Bypass	Retrospective study; treatment with liraglutide 3mg	Patients between the age of 18 and 60 with IWL defined as <50% EWL or RW defined as >10kg weight increase after NADIR after primary or revisional bariatric surgery	In months: 54.10 ± 31.75	12 months	% TWL liraglutide after primary surgery 6 months 5.95 ± 6.04 12 months 7.04 ± 7.53 No p- values mentioned, only p = 0.036 for 6 months vs. 12 months after liraglutide TWL % liraglutide after revisional surgery 6 months 6.08 ± 7.27 12 months 5.08 ± 9.05 No p- value mentioned, only p = 0.341 for 6 months vs. 12 months after liraglutide
2020 Edgerton et al. Patterns of Weight Loss Medication Utilization and Outcomes Following Bariatric Surgery	N=198 1 Bupropion 4 Bupropion/naltrexone 1 Bupropion/topiramate 49 GLP1 agonist 2 Liraglutide/topiramate 3 Lorcaserin 4 Metformin 1 metformine/phentermine 5 Metformin/topiramate 59 Phentermine 63 Phentermine/topiramate 6 Topiramate	N=197 125 SG 72 RYGB	Retrospective study; AOM after bariatric surgery	Adult patients >18 years with prior bariatric surgery who started AOM because of: WR defined as >20% WR after nadir, adequate weight loss and did not regain >20% weight but not satisfied and desired additional weight loss medication, and non-responders. Did not achieve goal weight loss.	In months: GS: 36.6 ± 19.5 RYGB: 102.7 ± 59.6	At least 2 months follow up after start AOM	%TWL per surgery: SG: 6.46 RYGB 9.12 (p = <0.01) % TWL per single medication Ph + T 9.8 ± 5.6 (p = <0.01) Ph 4.5 ± 3.7 GLP-1 7.7 ± 6.0 (p = 0.03) Other 6.2 ± 4.8
2020 Horber et al. Reversal of Long-Term Weight Regain After Roux-en-Y Gastric Bypass Using Liraglutide or Surgical Revision. A Prospective Study	N=95 30 Dietary and lifestyle counselling after WR <10% 15 Endosurgery 34 Liraglutide 16 Surgical resizing of the gastric bypass pouch with additional implanting of a Fobi-ring	95 RYGB	Prospective cohort study; Patients with <10% WR after nadir were the control group with lifestyle counselling. >10% WR after NADIR was treated with liraglutide, endosurgery or resizing of the RYGB pouch	Patients aged >18 who had undergone a Roux-en-Y gastric bypass at least six years earlier and had weight regain of more than 10% from NADIR weight	Liraglutide started 9 ± 5 years post operation	24 months	Mean weight loss in kg after 24 months: liraglutide: 13 ± 8 (p = <0.01) Resizing with Fobi ring: 17 ± 7 (p = <0.01) Endosurgery: 3 ± 3 after 12 months (p = not mentioned
2019 Miras et al. Adjunctive liraglutide treatment in patients with persistent or recurrent type 2 diabetes after metabolic surgery (GRAVITAS): a randomised, double-blind, placebo-controlled trial	N=71 48 Liraglutide 23 Placebo	80 total at baseline, number at analysis not mentioned 19 VSG 61 RYGB	Randomised, double-blind, placebo-controlled trial; liraglutide 1.8mg once daily or placebo together with a reduced-calorie diet, of 500kcal per day deficit.	Patients ≥18 year with obesity or persistent or recurrent type 2 diabetes defined as HbA1C higher than 48mmol/mol (>6.5%) who had undergone RUGB or VSG at least 12 months before randomization	In years: Placebo: 3.8 ± 2.4 Liraglutide 3.8 ± 2.0	26 weeks	Mean changes in KG with 95% confidence interval at 26 weeks total Placebo: -0.87, -2.14 to 0.41 Liraglutide: -5.26, -6.15 to -4.38 (p = <0.01)
2019 Wharton et al. Liraglutide 3.0 mg for the management of insufficient weight loss or excessive weight regain post-bariatric surgery	117 Liraglutide	N=117 14 SG 53 RYGB 50 AGB	Retrospective cohort study; liraglutide 3.0 and caloric intake deficit of 500 kcal/day on their daily energy expenditure	Patients with weight regain after bariatric surgery	In years: 7.8 ± 5.7 post surgery	12 months	% weight change RYGB: -6.6 ± 7.1 AGB: -4.9 ± 5.6 SG: -3.6 ± 3.0 (p = not mentioned)
2018 Stanford et al. Weight Loss Medications in Older Adults After Bariatric Surgery for Weight Regain or Inadequate Weight Loss: A Multicenter Study	One or a combination of the following: 4 Bupropion 3 Bupropion/naltrexone 7 Liraglutide 5 Lorcaserin 14 Metformin 3 Naltrexone 10 Phentermine 1 Phentermine/topiramate 20 Topiramate 8 Zonisamide	N=35 11 SG 24 RYGB	Retrospective study; one or more AOM after bariatric surgery. Medication started at plateau weight or with WR	Patients 60 years or older whom were prescribed weight loss medication after bariatric surgery and had at least 12 months follow up. Patients were prescribed a mean of 2.2 AOM over treatment period.	In months: RYGB 38.5 ± 28.6 SG 26.2 ± 13.2	Not mentioned	Mean BMI change SG: -1.38 ± 1.49 RYGB -3.37 ± 2.83 (p = 0.04)
2018 Toth et al. Weight Loss Medications in Young Adults after Bariatric Surgery for Weight Regain or Inadequate Weight Loss: A Multi-Center Study	Quantity per medicine is not given Metformin Phentermine Topiramate	N=37 9 SG 28 RYGB	Retrospective cohort study; phentermine, topiramate or metformin after bariatric surgery. Medication started at plateau weight or with WR	Young adults (21-30) who started phentermine, topiramate or metformin after bariatric surgery.	In months: SG 20.1 ± 5.2 RYGB 62.6 ± 39.1	12 months	Median %TWL 12 months Metformin: -2.9 (p = 0.02) Phentermine: -7.7 (p = 0.20) Topiramate -7.2 (p = 0.32) Median weight change in % by surgery SG: -3.3 (IQR -1.5, -5.3) RYGB -8.1 (IQR -2.6, -17.5)
2018 Rye et al. Efficacy of High-Dose Liraglutide as an Adjunct for Weight Loss in Patients with Prior Bariatric Surgery	20 Liraglutide	N=20 7 SG 7 RYGB 3 VBG 3 AGB	Retrospective chart review; liraglutide 3.0 and caloric intake deficit of 500kcal/day on their daily energy expenditure	Patients aged 18–65 with weight regain defined as >10% weight regain from NADIR or inadequate weight loss defined as <20% and BMI ≥30	Not mentioned	28 weeks	Median weight loss at 28 weeks: 9.7% (IQR 7.8-13.9%)
2017 Nor Hanipah et al. Efficacy of adjuvant weight loss medication after bariatric surgery	N=209 18 Lorcaserin 10 Naltrexone/bupropion 156 Phentermine 25 phentermine/topiramate	N=209 52 SG 126 RYGB 21 LGB 4 gastric plication 6 revisional bariatric surgery	Retrospective chart review; start AOM after WR or IWL	Patients >18 with <50% excess weight loss or regained at least 5% of their nadir weight despite dietary counselling and behavioral and lifestyle changes after bariatric surgery who were placed on weight loss medication for a minimum of 3 months	In months: 38, IQR 24-63	12 months	%TWL 12 months: RYGB: 2.8 (p = 0.01) SG:.3 (p = 0.02) LGB: 4.6 (p = 0.02)
2017 Stanford et al. The utility of weight loss medications after bariatric surgery for weight regain or inadequate weight loss: A multi-center study	75 Bupropion 123 Metformin 121 Phentermine 194 Topiramate 65 Zonisamide This study does not mention what combinations are used	N=319 61 SG 258 RYGB	Retrospective study; AOM after bariatric surgery	Patients who underwent RYGB or SG and received subsequent weight loss medications	In months RYGB 59.3 ± 36.7 SG 23.2 ± 15.3	12 months	% TWL 12 months SG: -4.3 ± 5.7 RYGB: -8.3 ± 8.1 (p = <0.01)
2016 Gorgojo-Martínez et al. Effectiveness and tolerability of liraglutide in patients with type 2 diabetes mellitus and obesity after bariatric surgery For this review, only the patients with bariatric surgery are considered	15 Liraglutide	N=15 1 SG 8 RYGB 3 BPD 2 VBG 1 LGB	Retrospective cohort study; liraglutide prescribed for additional weight loss or to combat DM2	Patients older than 18 without postsurgical remission of DM2, new-onset DM2 after BS, suboptimal weight loss defined as BMI higher than 35km/m² or %EWL lower than 50%) and weight regain higher than 5% after nadir.	In years 5.2 IQR 2.4 - 9.2	24 months	Weight drop in the BS group 24 months: 106.0 ± 7.2 to 102.6 ± 6.9 kg
2015 Schwartz et al. Pharmacotherapy in Conjunction with a Diet and Exercise Program for the Treatment of Weight Recidivism or Weight Loss Plateau Post-bariatric Surgery: a Retrospective Review	N=65 52 Phentermine 13 Phentermine/topiramate	N=65 51 RYGB 14 LGB	Retrospective review; start phentermine or phentermine/topiramate after bariatric surgery	Patients with a BMI of 27-29.9kg/m² with at least one co-morbidity including diabetes mellitus, medication controlled hypertension, hypercholesterolemia and/or obstructive sleep apnea; or a BMI>30 kg/m² without co-morbidities who have had bariatric surgery.	Not mentioned	90 days	KG EWL 90 days: Phentermine: 6.3 CI 4.25-8.44 Phentermine/topiramate: 2.8 CI 1.08-6.54

The 25 included studies vary in design. 19 are retrospective studies (13–30), five are randomized, double-blind, placebo-controlled trials (31–35), including one that utilized a two-way crossover design (31) and one prospective cohort study (36). The types of bariatric surgeries in these trials also varied. The most common procedure is RYGB (13, 15–29, 31, 32, 34–36), followed by SG (13–15, 17–19, 21–30, 34–36), and gastric banding (13, 16, 18, 19, 22–24, 27–29). Other bariatric procedures include biliopancreatic diversion (BPD) (18, 29), gastric plication (28) and revisional SADI (23).

The AOM investigated across the included studies vary considerably. GLP-1 RAs, particularly liraglutide and semaglutide, were the most frequently prescribed agents (13–15, 19–25, 27, 29–36). Dulaglutide was investigated only in the study by Jirapinyo et al. (23). Three studies investigated the additional effect of the dual GLP1-GIP agonist tirzepatide (14, 17, 30). Other investigated medication include phentermine, either as monotherapy or in combination with topiramate (15, 16, 20, 21, 25, 26, 28). Another study reported on the combination of phentermine with metformin (22). Topiramate as monotherapy was also quite common (15, 18, 21, 25). It is also given in combination with sibutramine (30), metformin, liraglutide, and bupropion (15). The other studies described the results of metformin (15, 20, 25, 26), bupropion and naltrexone as either monotherapy or in combination with each other (15, 20, 25, 28), sibutramine (18), orlistat (18, 21), zonisamide (25) and lorcaserin (15, 20, 25, 28). Two studies compared medication versus new bariatric intervention after weight regain after RYGB or SG (30, 36).

The placebo-controlled studies compared the effectiveness of liraglutide versus placebo in patients with weight regain after RYGB (32), patients with poor weight loss response after RYGB or SG (34), or persisted or recurrent type 2 diabetes after RYGB or vertical SG (VSG) (35). In these studies weight loss on liraglutide post RYGB was respectively 8.8% TWL after 12 months (32), 9.49 ± 5.07kg after 6 months (34) and -5.26kg with a confidence interval (CI) ranging from -6.15 to -4.38 kg after 6 months.

One study compared liraglutide to placebo six weeks after conversion RYGB (cRYGB). After 12 months, this trial demonstrated a %TWL of 24.15 ± 2.35 on cRYGB in combination with liraglutide versus 22.70 ± 2.13 on cRYGB with placebo (33). The double-blind, two-way crossover trial compared the combination of transoral outlet obstruction endoscopy (TORe) with liraglutide or placebo in patients with weight regain after RYGB. TORe utilizes an endoscopic suturing or plication device to reduce the diameter of the gastrojejunal anastomosis and/or the gastric pouch, thereby emulating the anatomical modifications achieved through surgical revision (20). The intervention of saline and liraglutide switched after 12 months. After 12 months the liraglutide and TORe group lost 21.69 ± 1.64%TWL while the placebo group lost 14.37 ± 2.58. After 24 months, when all participants have had both 12 months of placebo and 12 months of liraglutide, the group that started with liraglutide had 15.74 ± 5.84%TWL while the other group lost 11.04 ± 4.36%TWL (31).

Adverse events were reported in all five randomized controlled trials. Patients in the liraglutide group experienced more adverse events than those in the placebo group. The most commonly reported side effects were gastrointestinal in nature, with nausea, diarrhea, and constipation being the most frequently observed (31–35).

The prospective cohort study investigated the difference in weight loss between AOM and a novel bariatric intervention. These interventions were split among patients who had undergone a RYGB at least six years prior and had subsequently regained more than 10% of their weight after reaching their nadir. The interventions compared were liraglutide, endoscopic surgery, and resizing of the RYGB pouch using a Fobi ring. After 24 months, patients treated with liraglutide experienced a weight loss of 13 ± 8 kg. Those who received a Fobi ring lost 17 ± 7 kg, while patients who underwent endoscopic surgery lost 3 ± 3 kg (36).

Another study compared weight loss outcomes in patients with a history of SG that, due to weight regain, were treated with either semaglutide, tirzepatide, or a revisional ESG (rESG). After 24 months, the %TWL was 9.2 ± 7 in the group treated with AOM, compared to 13.4 ± 3.7 in the group that underwent rESG (30).

The retrospective cohort studies have investigated the effects of various AOMs after bariatric surgery when administered in cases of weight regain or insufficient weight loss following RYGB, SG and adjustable gastric banding (AGB). Several of these studies have reported the weight-related outcomes per medication. Semaglutide, for instance, was evaluated in the study by Medhati et al., which demonstrated a %TWL of 13.6 ± 10.3 following RYGB and 11.4 ± 9.8 following SG after one year of treatment. Jamal et al. reported a percentage weight change of −10.3 ± 5 after six months of use in individuals with a history of SG. Murvelashvili found an average %TWL of 12.92 ± 1.17 after one year of semaglutide use, with greater efficacy in RYGB patients (15.83 ± 2.48) compared to those with SG (12.36 ± 1.8) or AGB (8.64 ± 2.53).

Liraglutide was investigated by Murvelashvili et al., who found a mean %TWL of 8.77 ± 1.23 after 12 months, with no significant differences between RYGB, SG, or AGB. Muratori et al. reported a BMI reduction of 5.0 ± 6.4 in RYGB patients, 7.6 ± 5.8 in those with LGB, and 5.6 ± 6.9 in patients with an SG. Elhag et al. compared liraglutide use following primary versus revisional bariatric procedures, reporting a %TWL of 7.04 ± 7.53 after one year in the primary group and 5.08 ± 9.05 in the revisional group. Wharton et al. reported a percentage weight change after one year of liraglutide in patients with an RYGB of -6.6 ± 7.1, in patients with an AGB of -4.9 ± 5.6, and in patients with SG of -3.6 ± 3.0. Rye et al. found a mean weight loss of 9.7% (IQR 7.8–13.9%) after 28 weeks of liraglutide treatment. Gorgojo-Martínez et al. reported a reduction in body weight from 106.0 ± 7.2 kg to 102.6 ± 6.9 kg after 24 months of liraglutide therapy.

Non-specified GLP-1 RAs were examined by Edgerton et al., who found a %TWL of 7.7 ± 6.0 after an undefined treatment duration. Tirzepatide was mentioned in the study by Stoll et al., showing a 12.0 ± 3.4% weight reduction after six months. Jamal et al. reported a weight change of −15.5 ± 6.3% after six months in patients with prior SG. Topiramate was investigated by Boger et al., who reported a weight loss of 3.2 kg after 24 months. Toth et al. found a %TWL of 7.2 after 12 months of use. The combination of topiramate/sibutramine was studied by Boger et al., reporting a weight loss of 6.1 kg after 24 months. The phentermine/topiramate combination was evaluated by Edgerton et al., who found a %TWL of 9.8 ± 5.6 after an undefined period. Schwartz et al. reported an excess weight loss (EWL) of 2.8 (CI 1.08–6.54) after 90 days. Phentermine alone was studied by Edgerton et al., showing a %TWL of 4.5 ± 3.7 after an undefined treatment duration. Toth et al. found a %TWL of −7.7 after 12 months, while Schwartz et al. reported an EWL of 6.3 (CI 4.25–8.44) after 90 days. Orlistat was investigated by Boger et al., who reported a TWL of 2.7 kg after six months. When used in combination therapy, it resulted in a weight reduction of 3.9 kg. Metformin was studied by Toth et al., who found a %TWL of 2.9 after 12 months of treatment.

Dharmaratnam et al. investigated if there was a difference in the effectiveness of orlistat, phentermine, liraglutide, topiramate as monotherapy or in combination with each other in patients with insufficient weight loss, weight regain <10% or weight regain ≥10%. They found a %TWL in the insufficient weight loss group of 0.7 ± 4.2, in the weight regain <10% group of 0.7 ± 4.6 and in the weight regain ≥10% group of 1.8 ± 4.3. Stanford et al. and Nor Hanipah et al. investigated the difference in weight loss after starting AOM between RYGB, SG and LGB. The effect of lorcaserin, naltrexone/bupropion, phentermine, phentermine/topiramate and bupropion, metformin, phentermine, topiramate, zonisamide were investigated. After a follow-up of 1 year, Nor Hanipah et al. found a %TWL in patients with a RYGB of 2.8, in patients with SG of 0.3 and in patients with LGB of 4.6. Stanford et al. found a %TWL of 4.3 ± 5.7 in patients with SG and -8.3 ± 8.1 in patients with RYGB.

This overview indicates that AOM after bariatric surgery are primary studied in case of insufficient weight loss or weight regain.

The most commonly investigated AOMs are GLP1-RAs and the dual agonist tirzepatide. These GLP-1 RAs appear to promote clinically meaningful weight loss in patients experiencing weight regain or insufficient weight loss after bariatric surgery. Liraglutide and semaglutide were the most commonly investigated agents, with %TWL outcomes often ranging between 8% and 15%, especially following RYGB (13–15, 19–25, 27, 29–36). Semaglutide and liraglutide demonstrated consistent effects across different surgical types (18, 23, 25, 28, 29, 32, 34, 35).

In contrast, non-GLP-1 RA medications such as phentermine, topiramate, orlistat, and bupropion/naltrexone showed more variable and generally lower weight loss, usually less than 10% TWL (15, 16, 18, 20–22, 25, 26, 28), which is in line with their efficacy in patients without bariatric surgery. Combination therapies outperformed monotherapy in some cases but did not consistently matched the efficacy of GLP-1 RAs (15, 16, 20, 21, 25, 28).

Some studies compared AOM to revisional interventions. While revisional surgeries like pouch resizing or endoscopic procedures often resulted in slightly greater weight loss (30, 33, 36), liraglutide produced comparable outcomes in certain cohorts, suggesting a viable non-surgical alternative, especially for RYGB patients (30, 36).

Most studies were retrospective cohorts and had additional limitations such as heterogeneity in AOM type, dosage, initiation timing and follow-up duration (13–30). Nonetheless, the real-world settings enhance the applicability of the findings. Some studies also noted improvements in metabolic parameters, including HbA1C reductions, reinforcing the dual benefit of GLP-1RAs for weight and glycemic control (29, 35).

Future studies on the use of AOM after bariatric surgery should focus on long-term results, additional health effects and side effects. In addition, the addition of directly AOM after bariatric surgery in those with very high initial body weight or severe comorbidity should also be investigated.

## Bariatric surgery and probiotics

We identified six articles that investigated the use of prebiotics in combination with bariatric surgery and that reported weight loss outcomes. Characteristics and results of these articles can be found in **Table 3**. Several other clinical studies and randomized controlled trials investigated effects other than weight loss of probiotics after bariatric surgery. These studies are not included in this narrative review.

**Table 3:** Gastric surgery and probiotics.

Study	Drug	Operation	Intervention	Patient profile	Time since surgery	Results
2024 Melali et al. Impact of Probiotics on Gastrointestinal Function and Metabolic Status After Roux-en-Y Gastric Bypass: A Double-Blind, Randomized Trial	N= 135 68 Probiotics (specifics are not mentioned) 67 Placebo	135 RYGB	Randomized, double-blind trial; probiotics versus placebo For 3 months	Patients 16 to 60 years of age wit BMI ≥ 40kg/m² or 35≤BMI ≤ 40kg m² with pertinent comorbidities	Probiotics or placebo started 1 week after surgery	KG TWL 6 months Probiotics: 35.31 ± 3.21 Placebo: 35.47 ± 2.98 (p = 0.37)
2024 Potrykus et al. Preoperative Multistrain Probiotic Supplementation Does Not Affect Body Weight Changes or Cardiometabolic Risk Factors in Bariatrics: Randomized, Double-Blind, Placebo-Controlled Clinical Trial	N=48 22 Probiotics (a mixture containing Bifidobacterium bifidum W23, Bifidobacterium lactis W51 and W52, Lactobacillus acidophilus W37, Levilactobacillus brevis W63, Lacticaseibacillus casei W56, Ligilactobacillus salivarius W24, Lactococcus lactis W19, and Lactococcus lactis W58 in daily dose 2 × 109 CFU) 26 Placebo	SG OAGB	Randomized, double-blind, placebo-controlled clinical trial; preoperative probiotic supplementation versus placebo. 4 capsules a day before surgery	Patients over 18, Caucasian race and eligible for bariatric surgery based on IFSO guidelines.	Probiotics or placebo started 12 weeks before surgery	% TWL 6 months Probiotic: 30.4 ± 11.5 Placebo: 26.5 ± 8.8 (p = 0.23)
2021 Ramos et al. Effects of Lactobacillus acidophilus NCFM and Bifidobacterium lactis Bi-07 Supplementation on Nutritional and Metabolic Parameters in the Early Postoperative Period after Roux-en-Y gastric Bypass: a Randomized, Double-Blind, Placebo-Controlled Trial	N=71 38 Probiotics (a mixture containing 5 billion Lactobacillus acidophilus NCFM^®^ Strain, 5 billion Bifidobacterium lactis Bi-07^®^) 33 Placebo	71 RYGB	Randomized, double-blind, placebo-controlled trial; Probiotics versus placebo for 90 days	Adult candidates with a BMI of ≥ 35kg/m² and did not use antibiotics 4 weeks prior to the beginning of the study	Probiotics or placebo started seventh postoperative day	% EWL 12 weeks Probiotics: 47.94 (9.96) Placebo: 50.87 (12.73) (p = 0.29)
2021 Calikoglu et al. The Metabolic Effects of Pre-probiotic Supplementation After Roux-en-Y Gastric Bypass (RYGB) Surgery: a Prospective, Randomized Controlled study	N=30 14 Prebiotic (Beneo Orafti Sinergy-1^®^: inulin+oligofructose) 16 probiotic (conventional yogurt)	30 RYGB	Prospective, parallel randomized, controlled trial; probiotics versus placebo for 6 months	Patients who underwent laparoscopic RYGB due to morbid obesity	Prebiotics or probiotics started two weeks after surgery	% EWL 6 months Pro-probiotics: 56.23 ± 20.06 Probiotics: 56.78 ± 13.30 (p = 0.928)
2018 Karbaschian et al. Probiotic Supplementation in Morbid Obese Patients Undergoing One Anastomosis Gastric Bypass-Mini Gastric Bypass (OAGB-MGB) Surgery: a Randomized Double-Blind, Placebo-controlled, Clinical Trial	N=46 23 Probiotics (a mixture containing Lactobacillus casei (3.5 × 109 CFU/g), Lactobacillus rhamnosus (7.5 × 108 CFU/g), Streptococcus thermophilus (1 × 108 CFU/g), Bifidobacterium breve (1 × 1010 CFU/g), Lactobacillus acidophilus (1 × 109 CFU/g), Bifidobacterium longum (3.5 × 109 CFU/g), and Lactobacillus bulgaricus (1 × 108 CFU/g)) and 38.5 mg fructo-oligosaccharide) 23 Placebo	46 OAGB	Placebo-controlled, double-blind, randomized clinical trial; probiotics verses placebo	Woman between 18 and 60 years old with BMI ≥40kg/m² or 40 > BMI>35 kg/m² with comorbidities	Probiotics or placebo started 4 weeks before surgery until 12 weeks after surgery	% EWL 16 weeks Probiotics: 46.82 ± 12.69 Placebo: 36.34 ± 12.66 (p = 0.01)
2018 Sherf-Dagan et al. Probiotics administration following Sleeve Gastrectomy surgery: a randomized double-blind trial	N=80 40 Probiotics 40 Placebo	80 SG	Randomized double-blind, placebo-controlled trial; probiotics versus placebo for 6 months	Patients between 18 and 65 years with NAFLD, BMI >40 kg/m² or BMI >35kg/m² wit co-morbidities	Not mentioned	% EWL 6 months Probiotics: 61.2 ± 14.1 Placebo: 64.0 ± 12.4 (p = 0.54)

The six studies included in this review had a similar design, all were randomized controlled trials. These trials involved a combined population of 410 patients, of whom 205 received a probiotic intervention (37–42).

Five of the studies compared probiotics to placebo (37–39, 41, 42), while one study investigated prebiotics versus conventional yogurt (40). In two studies, treatment with probiotics or placebo was initiated prior to bariatric surgery (38, 41). In three studies, the intervention commenced after surgery (37, 39, 40), whereas one study did not specify the timing of the intervention (42).

The most common surgical procedure among the included trials was RYGB (37, 39, 40), followed by one anastomosis gastric bypass (OAGB) (38, 41) and SG (38, 42).

The follow-up duration ranged from 12 weeks to six months. Four studies reported %EWL as the primary outcome. In the probiotics groups, %EWL ranged from 61.2 ± 14.1 to 46.8 ± 12.7, while in the placebo groups it ranged from 64.0 ± 12.4 to 36.3 ± 12.7. One study reported %TWL, which was 30.4 ± 11.5 in the probiotics group and 26.5 ± 8.8 in the placebo group after six months. The final study reported total weight loss in kilograms, with a loss of 35.3 ± 3.2 kg in the probiotics group and 35.5 ± 3.0 kg in the placebo group.

None of the reviewed trials found a statistically significant difference in weight loss between the probiotics and placebo groups. Although these findings suggest that probiotic supplementation, whether initiated before or after bariatric surgery, does not significantly affect total weight loss, a systematic review of four trials by Zhang et al. reported that probiotic intake at the time of bariatric surgery may have a beneficial effect on waist circumference (43).

Potrykus et al. evaluated besides weight loss also the effect on glycemic control, lipid profiles, liver enzyme levels, iron status, and vitamin levels. Across all the parameters, no significant differences were identified between the probiotic and placebo groups (38).

A systematic review examined the effects of probiotics and synbiotics on small intestinal bacterial overgrowth (SIBO) and other gastrointestinal symptoms (GIS). This review included five studies with a combined total of 396 patients and follow-up durations ranging from two weeks to six months. Pooled meta-analysis revealed no significant differences between probiotics, synbiotics, and placebo in alleviating SIBO or GIS symptoms (44). Although gastrointestinal complaints are common following bariatric procedures and may be influenced by microbial dysbiosis, current evidence does not support the efficacy of microbial supplementation in this context. The variability in probiotic strains, dosages, treatment durations, and diagnostic criteria for SIBO across studies may have contributed to these equivocal results (44).

A prospective randomized trial assessed the incidence of gallstone formation following SG or OAGB in patients receiving probiotics, digestive enzymes, or ursodeoxycholic acid (UDCA). These interventions commenced three days postoperatively and were continued for six months. After six months, fewer gallstones or biliary sludge were observed in the groups receiving UDCA or probiotics compared to the group receiving digestive enzymes; however, this difference did not reach statistical significance (45). This indicates that the study was underpowered, since the efficacy of UDCA to prevent gallstone formation after bariatric surgery has been demonstrated repeatedly (46). An adequately powered study should be performed to evaluate the potential of probiotics for the prevention of gallstones.

Additionally, a study explored whether the addition of prebiotics to the diet of postmenopausal women who had undergone RYGB could enhance calcium absorption. No significant differences were found between the prebiotic and placebo groups. The wide confidence intervals suggest a potentially variable effect of short-chain fermentable fiber (SCF) across this population (47). Again, the small sample size precludes clear conclusions and an adequately powered study is warranted.

Overall, the studies did not show a consistent meaningful effect of probiotics and prebiotics on weight loss after bariatric surgery. The effect on metabolic parameters was also limited. The small sample sizes of the studies and heterogeneity in interventions makes it impossible to draw strong conclusions. Two systematic reviews came to similar conclusions (43, 48).

While the meta-analysis from Zhang et al. (43) identified statistically significant reduction in waist circumference at 12 months of -4.21 cm in favor of the probiotics group, the meta-analysis of Wang et al. (48) did not found a statically significant difference. It did however found a statistically significant difference in weight and BMI in favor of the probiotics group, this was however not found by the first meta-analysis. Similarly, Wang et al. (48) observed a small but statistically significant differences the liver enzyme aspartate aminotransferase and vitamin B12 levels in favor of probiotic use. These effects however, were modest in magnitude and did not extend to other related markers, including alanine aminotransferase, Gamma-GT, or additional micronutrients.

In this review we discuss interventions targeting gut microbiome separately. However, we should realize that both bariatric surgery and several AOM such as GLP1-RAs and metformin do have clear influences on gut microbiome composition and related metabolites. These alterations may contribute to the observed effects of bariatric surgery and AOM.

Bariatric surgery and GLP-1 receptor agonists induce significant changes in gut microbiota, often increasing beneficial bacteria such as *Akkermansia muciniphila*, short-chain fatty-acid-producing Bacteria, and other taxa linked to improved metabolic outcomes, while reducing pro-inflammatory species. Although responses vary across individuals, studies and specific treatments, bariatric surgery is generally considered to have a positive effect on gut microbiota (49).

Liraglutide promotes genera linked to improved metabolism, and dulaglutide and semaglutide produce distinct changes in specific beneficial taxa and microbial diversity. These alterations may support long-term metabolic health, aid diabetes management, and help maintain healthy body weight (50). Due to the heterogeneity in probiotic strains used, variations in the timing of interventions, and the short follow-up periods, it is challenging to draw definitive conclusions in regards to weight loss.

## Clinical considerations

Although current evidence suggests that GLP-1 RAs and other AOMs can enhance weight loss after bariatric or endoscopic procedures, several clinically relevant issues remain unresolved. Data on weight trajectories after cessation of AOMs in patients post-bariatric surgery are lacking; however, extrapolation from non-surgical obesity trials suggests substantial weight regain after discontinuation, indicating that chronic treatment may be required to maintain weight loss (51).

The implications of prolonged AOM use in patients who have undergone bariatric surgery are insufficiently studied. Most available studies evaluate outcomes over relatively short follow-up periods, whereas obesity is a chronic disease that may necessitate sustained pharmacological therapy. If ongoing treatment is required, this raises important questions regarding long-term safety, tolerability, adherence, and cost-effectiveness in a post-bariatric population, particularly given the altered gastrointestinal anatomy and potential differences in drug absorption. At present, these considerations remain largely unaddressed in the existing literature.

In addition, differences between outcomes reported in clinical trials and those observed in routine clinical practice warrant attention. RCTs typically involve carefully selected patient populations, standardized dosing protocols, and intensive follow-up, which may not reflect real-world settings where access to medication, reimbursement policies, long-term adherence, and follow-up intensity vary considerably. Furthermore, most real-world studies are retrospective and heterogeneous in terms of patient selection, timing of treatment initiation, and choice of pharmacotherapy, limiting direct comparisons and generalizability.

Taken together, these unresolved issues highlight the need for prospective studies with longer follow-up to better define the durability of weight loss, optimal timing of treatment initiation, and the long-term role of AOM after bariatric or endoscopic interventions. Such studies are essential to guide personalized, evidence-based treatment strategies in everyday clinical practice.

## Conclusion

Available evidence suggests that combining endoscopic interventions with AOMs, particularly GLP-1RAs, is associated with greater and more clinically significant weight loss than ESG alone. Reported outcomes indicate that adjunctive AOM following ESG improves weight loss outcomes from 15 –21% TWL with ESG alone to 17 – 25% TWL when combined with GLP-1 RAs (9–12). However, ESG combined with other AOMs does not result in significantly greater weight loss than ESG alone (12).

In patients with insufficient weight loss or weight regain after bariatric surgery, GLP-1RAs and the dual GIP/GLP-1RA tirzepatide are associated with clinically meaningful weight loss. With a TWL between 8-15% in the GLP-1RA group (13–15, 19–25, 27, 29–36) and a TWL between 12-15% with the use of tirzepatide (14, 17, 30). By contrast, non-GLP-1 medications typically results in less than 8% TWL and demonstrate more modest and variable effects (15, 16, 18, 20, 21, 25, 26, 28).

Probiotic supplementation during or shortly after bariatric surgery has not demonstrated consistent or clinically relevant effects on postoperative weight loss, and no specific strain or patient subgroup has been identified as clearly beneficial. However, due to the small sample sizes, short follow-up periods and heterogeneity in interventions it is not possible to draw definite conclusions (37–42).

Overall, current evidence suggests thatGLP-1RAs appear to be the most effective AOM in combination with ESG to optimize weight loss. It also appears to be the most valuable for patients with insufficient weight loss or weight regain after bariatric surgery, whereas the role of other AOMs and probiotics remains uncertain.

However, long-term results of AOM use after bariatric or endoscopic procedures is insufficiently studied. Data on weight changes after discontinuation of AOM is lacking. As obesity is a chronic disease, sustained or long-term AOM treatment may be required to maintain weight loss, raising questions regarding long-term safety, adherence, cost and real world feasibility in post-bariatric populations. Further research should prioritize these questions.

Due to the heterogeneity of the topic and the limited number of existing studies, we opted to conduct a narrative review rather than a systematic review. The variability in the available literature, combined with the scarcity of rigorous research, makes it challenging to synthesize findings through a systematic approach. A narrative review enables a more flexible and comprehensive examination of the subject, acknowledging both its complexity and the gaps in the current evidence base. Therefore, this approach enables a broader discussion of the topic, while highlighting areas for future research.
